# Standardization of Quantitative Plaque-Based Viral Assays for Orthoflavivirus Cacipacoré

**DOI:** 10.3390/v17101355

**Published:** 2025-10-10

**Authors:** Marielena Vogel Saivish, Natalia I. O. da Silva, Madeline R. Steck, Rafael E. Marques, Mauricio L. Nogueira, Shannan L. Rossi, Nikos Vasilakis

**Affiliations:** 1 Laboratórios de Pesquisas em Virologia, Departamento de Doenças Dermatológicas, Infecciosas e Parasitárias, Faculdade de Medicina de São José do Rio Preto, São José do Rio Preto 15090-000, SP, Brazil; marielenasaivish@gmail.com (M.V.S.); mauricio.nogueira@edu.famerp.br (M.L.N.); 2 Laboratório Nacional de Biociências, Centro Nacional de Pesquisa em Energia e Materiais (CNPEM), Campinas 13083-100, SP, Brazil; rafael.marques@lnbio.cnpem.br; 3 Department of Pathology, University of Texas Medical Branch, Galveston, TX 77555-0609, USA; naolivei@utmb.edu (N.I.O.d.S.); mrsteck@utmb.edu (M.R.S.); 4 Institute for Human Infection and Immunity, University of Texas Medical Branch, Galveston, TX 77555-0610, USA; 5 Center for Vector-Borne and Zoonotic Diseases, University of Texas Medical Branch, Galveston, TX 77555-0609, USA; 6 World Reference Center for Emerging Viruses and Arboviruses, University of Texas Medical Branch, Galveston, TX 77555-0609, USA

**Keywords:** genus *Orthoflavivirus*, family *Flaviviridae*, emerging infectious diseases, Cacipacoré virus

## Abstract

Cacipacoré virus (CPCV) is an understudied orthoflavivirus with significant gaps in research that hinders our understanding of its ecology, host range, and potential public health impact. A notable barrier to filling this gap is the absence of standardized methodologies for viral quantification, such as plaque-forming and focus-forming assays. This short communication outlines the development of optimized protocols for CPCV titration in two cell lines, Vero CCL-81 and BHK CCL-10, using several overlay and time point conditions. These methodologies address the need for robust quantitative tools to advance research on CPCV and its implications for human and animal health, fostering progress in the surveillance and understanding of this underexplored arbovirus.

## 1. Introduction

Cacipacoré virus (CPCV), a member of the genus *Orthoflavivirus* within the family *Flaviviridae*, was first isolated in 1977 from the blood of a black-faced antthrush (*Formicarius analis*) in the Brazilian Amazon [1]. Despite its classification within the Japanese encephalitis virus (JEV) serocomplex, CPCV remains severely understudied compared to its well-known orthoflavivirus relatives, such as dengue and Zika viruses. Initial studies suggested a complex transmission cycle involving diverse hosts and potential vectors, including mosquitoes (*Aedes aegypti, Culex* sp. and possible *Anopheles* sp.) [2] and birds [1,3], although more studies are required to tease apart these relationships. This lack of foundational knowledge hinders efforts to assess the epidemiological relevance and potential threat to both public and veterinary health of CPCV.

**Figure 1:**
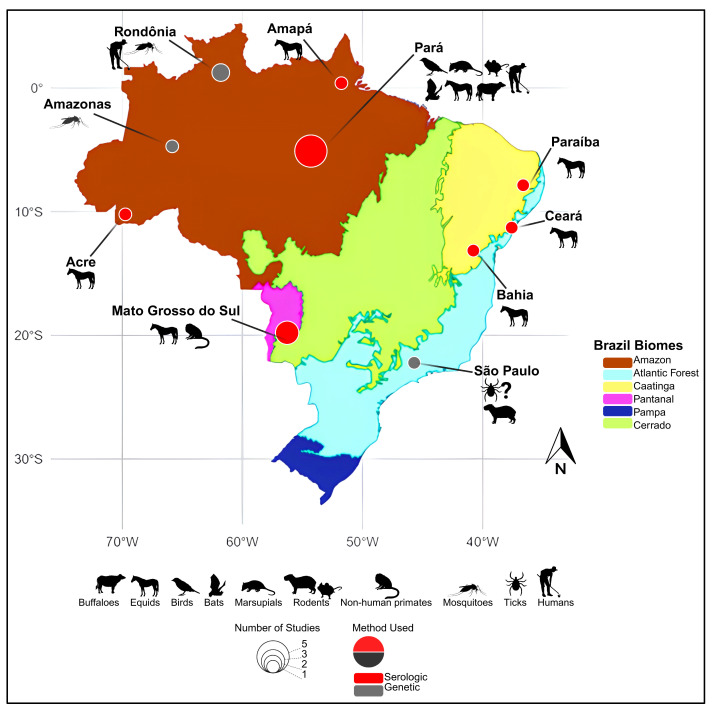
Geographical distribution of historical Cacipacoré virus (CPCV) detections in Brazil. The map highlights regions where CPCV has been identified either through serological or molecular methods. Red circles indicate locations with serological evidence, while gray circles represent sites with confirmed genetic detection. The diameter of each circle is proportional to the number of published reports associated with that location. Amazon (light green), Caatinga (yellow), Cerrado (dark green), Pantanal (blue), Atlantic Forest (light brown), and Pampa (gray). Base map adapted from official IBGE biome maps (available at: https://geoftp.ibge.gov.br/informacoes_ambientais/estudos_ambientais/biomas/mapas/biomas_5000mil.pdf, accessed on 26 January 2025 and https://www.ibge.gov.br/apps/biomas/#/home, accessed on 26 January 2025) and Saivish et al. [5].

Emerging evidence underscores the ecological and geographical adaptability of CPCV. Although its presence has only been documented within Brazil, it is important to recognize the country’s continental size and environmental diversity, encompassing multiple independent biomes [4,5]. CPCV has been detected in diverse ecotypes (Figure 1), including the Amazon rainforest [1,3,6,7,8,9], urban areas near the Amazon [2], and biomes as distinct as the semi-arid Caatinga (xeric shrubland and thorn forest) in northeastern Brazil [10,11], the Cerrado (tropical savanna), and the Pantanal wetlands in the southern-central region [12,13,14]. Furthermore, the virus was detected in the southeastern Atlantic Forest, over 2000 km from the Amazon, where it was isolated from engorged ticks feeding on a viremic capybara [15]. This broad distribution across diverse ecosystems, including both natural and urbanized settings, underscores the virus’s capacity to adapt to varied ecological niches and raises important questions regarding its potential as a zoonotic threat [2].

To date, no studies have established standardized methods to quantify CPCV infectivity using plaque- or focus-forming unit (PFU or FFU, respectively) assays. This hinders studies on viral replication dynamics, fitness, antiviral screening, and phenotypic assessment of CPCV strains, all of which require reproducible quantitative tools. The absence of specific diagnostic and titration methodologies also complicates efforts to assess the true burden of the virus. Given the well-documented serological cross-reactivity among orthoflaviviruses, CPCV infections could be misdiagnosed as other orthoflaviviral diseases, although direct evidence of CPCV-specific cross-reactivity remains limited [5,6,16]. Viral quantification techniques, including the ability to quantify virus PFU and FFU titers, are indispensable for quantifying infectious viral particles, yet no such protocols exist for CPCV, highlighting a critical gap in the field.

Current detection of CPCV has relied on two main approaches. Molecularly, genus-specific orthoflavivirus RT-PCR/hemi-nested RT-PCR frameworks with sequencing have been used to identify CPCV RNA in field samples (including mosquito pools) and to genetically characterize virus from ticks, reflecting the broader reliance on pan-orthoflavivirus primer sets in surveillance contexts for neglected arboviruses [2,15]. Serologically, equine surveys in the Brazilian Pantanal employed screening ELISAs followed by confirmatory plaque-reduction neutralization tests (PRNT_90_) against multiple orthoflaviviruses and detected neutralizing antibodies to CPCV at low frequency, underscoring feasibility but also apparent rarity of exposure [13,17]. In line with WHO guidance and systematic reviews, PRNT remains the reference assay for orthoflavivirus serodiagnosis, albeit with recognized inter-laboratory variability that reinforces the need for standardized methods [18,19]. Historically, CPCV was also recovered by inoculation of newborn mouse brain, documenting early culture-based detection [3]. Together, these strands highlight a diagnostic landscape that is molecular/serological but lacks standardized, CPCV-specific infectivity titration protocols—a crucial gap.

This study addresses the need for standardized titration methods by presenting optimized protocols for CPCV quantification in Vero and BHK cell lines. By developing these methodologies, we aim to provide a foundation for future research on CPCV, thereby enabling more precise studies on host–pathogen interactions, viral kinetics, and the development of potential therapeutic research.

## 2. Materials and Methods

### 2.1. Safety Practices

All in vitro studies, including manipulation of cell cultures and viruses, were performed under biosafety level 2 laboratory conditions, according to the environmental, health and safety rules and regulations of the University of Texas Medical Branch at Galveston, Texas. All operations were carried out under strict aseptic conditions. Fixation methods for orthoflaviviruses were used here to render material non-infectious prior to staining. All experiments were performed under BSL-2 conditions at UTMB, in accordance with institutional biosafety risk assessments and NIH/CDC guidelines. CPCV has no documented human outbreaks or efficient human-to-human transmission, which supports BSL-2 classification in this context, although some institutions may adopt BSL-3 depending on local risk assessments.

### 2.2. Virus Strains and Cell Lines

African green monkey kidney (Vero) cells (CCL-81, ATCC, Manassas, VA, USA) (passages 160–179) and BHK (CCL-10, ATCC, Manassas, VA, USA) (passages 15–22) were grown in Dulbecco’s modified eagle medium (DMEM, Gibco, Grand Island, NY, USA), supplemented with 10% heat-inactivated fetal bovine serum (Gibco, Grand Island, NY, USA) and 1% penicillin-streptomycin solution (10^4^ U/mL and 10^4^ μg/mL solution, respectively) (PenStrep, Gibco, Grand Island, NY, USA) at 37 °C with 5% CO_2_. Cacipacoré virus strains BeAn 327600 and RP 962768 were obtained from the University of Texas Medical Branch’s World Reference Center for Emerging Viruses and Arboviruses (WRCEVA) and used to infect the cell lines. All viruses and cell cultures used in the present study tested negative for mycoplasma infection.

### 2.3. Titrations

Briefly, virus samples were assayed by 10-fold serial dilution in DMEM on Vero or BHK cell monolayers. After 1 h (hour), wells were overlaid with one of the overlay conditions described below. Following 3, 4, or 6 days incubation at 37 °C with 5% CO_2_, the overlay was removed, and monolayers were rinsed twice with sterile DPBS, and fixed for 1 h with ice-cold methanol/acetone (1:1). Detection of virus was visualized via plaque-forming unit assay or focus-forming unit assay, as detailed below (Table 1). Overlay concentrations of 0.2–0.8% were selected based on orthoflavivirus titration literature, where this range balances plaque clarity with monolayer preservation. Preliminary attempts with 1% agarose disrupted the monolayer during removal, and 1% CMC was not available in our laboratory at the time. Unless otherwise noted, each dilution was plated in duplicate wells (intra-assay technical replicates, n = 2). Where material permitted, the same virus stock was re-titrated in independent runs (n = 2) performed on different days using fresh cell passages and independent stock aliquots. For some conditions, only intra-assay duplicates were available due to sample constraints. An independent run is defined as a titration performed on a different day with a fresh cell passage and a separately thawed aliquot of the same virus stock.

### 2.4. Focus-Forming Unit Assay

FFU assays were performed as previously described [20,21], with modifications. Viral dilutions were inoculated onto Vero and BHK cell monolayers in 6-well plates, respectively. Following a 3, 4, or 6 days post-infection (d.p.i), plates were washed and fixed as described previously, then stained using the pan-orthoflavivirus 4G2 monoclonal antibody [22] and HRP-labeled goat anti-mouse secondary antibody (KPL, Gaithersburg, MD, USA). Detection was performed using KPL TrueBlue peroxidase substrate (SeraCare, Milford, MA, USA) according to manufacturer’s protocol. FFUs were counted manually, and viral titers were calculated as focus-forming units per milliliter (FFU/mL), accounting for the dilution factor and inoculum volume. The foci formed under the conditions were measured using the Mica microscope (Leica Microsystems, Wetzlar, Germany) and represent the range values of between the smallest and largest plaques observed. The original measurements, taken in micrometers, were converted to millimeters and rounded to two decimal places for consistency and precision. The complete protocol is described in detail in the Supplementary Materials.

### 2.5. Plaque-Forming Unit Assay

PFU assays were performed as previously described [23], with modifications. Infections were performed as described in Section 2.4 above. Following adsorption, the inoculum was removed, and cells were overlaid with one of the overlay conditions (Table 1) described before to restrict viral spread. Plates were then incubated for 3-, 4- or 6- days, after which monolayers were fixed then stained with 2% crystal violet to visualize plaques. Plates were rinsed with water and air-dried before plaque counting. Plaques were counted manually, and viral titers were calculated as plaque-forming units per milliliter (PFU/mL), accounting for the dilution factor and inoculum volume. We also measured the plaque size diameters under the conditions described in Table 2 and Table 3, following the same methodology outlined in Section 2.4. Measurements were performed using the Mica microscope (Leica Microsystems, Germany), with the results expressed in millimeters, derived from the conversion of micrometer measurements, and rounded to two decimal places for precision and consistency. The complete protocol is described in detail in the Supplementary Materials.

### 2.6. Image Acquisition

Images were acquired using the Mica microscope (Leica Microsystems, Germany), a high-resolution imaging system equipped with advanced mosaic and bright-field imaging functionalities. Whole-plate images of six-well plates were captured using the mosaic acquisition mode, enabling the generation of stitched composites that provide a comprehensive view of each plate. This approach ensured accurate visualization and documentation of all wells under standardized imaging conditions. For detailed examination of plaques and foci, bright-field imaging was employed to capture high-contrast images at appropriate magnifications, facilitating precise measurements of plaque and focus sizes. Raw acquired images were subsequently assembled into figure panels using the Canva platform (www.canva.com; Canva Pty Ltd., Surry Hills, Australia). For immunostained FFU panels, image contrast was increased to 100% and brightness decreased by 50 units to enhance foci visibility. PFU panels stained with crystal violet were presented without any adjustment to contrast or brightness.

## 3. Results

### 3.1. Comparison of Methylcelulose Overlays

**Figure 2:**
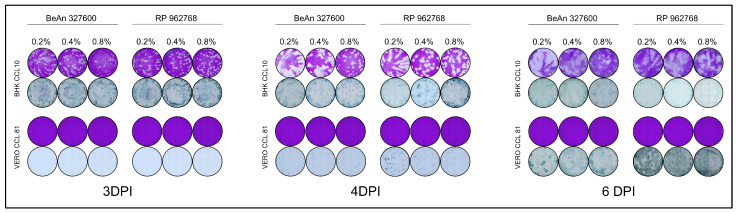
Parallel plaque and focus assays performed with three different concentrations of methylcellulose overlay in BHK CCL-10 and Vero CCL-81 cells. Representative plates shown.

To standardize the titration of CPCV strains, we evaluated three different concentrations of methylcellulose overlay (0.2%, 0.4%, and 0.8%) to identify the optimal conditions for visualization of plaques using crystal violet staining or colorimetric detection of infected cells based on antibody binding for the PFU and FFU assays, respectively (Figure 2). Vero and BHK cells were selected due to their wide use in arbovirus research, commercial availability, ability to consistently create uniform monolayers, and permissiveness to CPCV infection. We selected 3, 4, and 6 d.p.i. to evaluate the progression of viral replication and determine optimal conditions for plaque and focus assays that would not require extended incubation times, balancing early detection and late stages to ensure clear visualization while preserving monolayer integrity.

Plaques were visualized by crystal violet staining at 3, 4, and 6 d.p.i. under all three overlay conditions tested in BHK cells. Similarly, antibody-stained foci were easily visualized at 3 and 4 d.p.i. across all methylcellulose concentrations but became too large to individually distinguish at 6 d.p.i. (Figure 2). The measurement of the plaque size and the foci diameters can be seen in Table 2. As expected, both foci and plaques are larger at lower methylcellulose concentrations (0.2%) and become smaller and more contained at higher methylcellulose concentrations (0.8%). Based on these observations, we determined that PFU assays performed on BHK cells are most effective at 6 d.p.i. with an overlay consisting of 0.8% methylcellulose. For FFU assays, 3- or 4- d.p.i. are recommended for optimal visualization. It should also be considered that the lower methylcellulose concentrations of 0.2% and 0.4% appear to be more susceptible to slight movements that may occur within the incubator during the incubation period of the PFU or FFU assay, whereas the 0.8% concentration seems to be less affected by such plate movements.

Interestingly, while BHK cells showed visible CPE, Vero cells failed to produce plaques under similar conditions and methylcellulose overlay (Figure 1), despite previous reports of plaque formation at 8 d.p.i. in Vero cells (as described by Arbocat-Arbovirus Catalog [3]). To ensure incubation time was not insufficient for plaque formation, the incubation time was extended up to 11 d.p.i., but no plaques were observed. These results indicate that this cell line is not permissive to lytic plaque formation for either CPCV strains. In contrast, infection could be detected by antibody-detected foci. Foci were not visible at 3 d.p.i., small at 4 d.p.i., and more distinct at 6 d.p.i. in all methylcellulose concentrations tested (Figure 2, Table 1). Notably, no monolayer destruction or CPE was apparent at any of these time points. Based on these findings, we conclude that Vero are unsuitable for CPCV plaque assays. However, FFU assays may be feasible starting from 4 d.p.i., with more pronounced and distinguishable FFUs observed at 6 d.p.i.

### 3.2. Comparison of Low Melting Agarose Overlays

**Figure 3:**
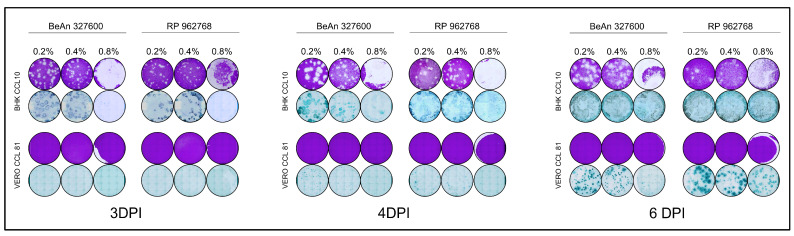
Plaque and focus assays performed with three different concentrations of low melting agarose overlay in BHK and Vero cells.

To optimize the potency assays of CPCV strains, we tested three concentrations of low-melting agarose overlay (0.2%, 0.4%, and 0.8%) to identify the most suitable conditions. The assays were conducted using Vero and BHK cell lines, with evaluations also performed at 3, 4, and 6 d.p.i. (Figure 3). In BHK cells, plaque formation was consistently observed at all three time points and under all overlay conditions, reinforcing that this cell line is permissive to both CPCV strains. However, the 0.8% agarose overlay presented challenges during fixation and removal, especially in the assays involving BHK, where even gentle and careful removals of the agarose overlay caused damage to the monolayer and consequent impact on the evaluation of the results (as shown in Figure 3). For this reason, this condition was considered inadequate for continuing the assays. FFUs were successfully visualized at 3 and 4 d.p.i. across 0.2% and 0.4% overlay concentrations, with better definition observed at these earlier time points. The concentration of 0.8% was also considered non suitable for FFU assay due to the damage during the overlay removal. Based on these findings, we determined that PFU assays performed in BHK cells are most reliable at 3 or 4 d.p.i. It is important to note that 3 or 4 d.p.i. plaques are small and are suitable for use in smaller well-plates, such as 24-well plates. However, if there is an interest in performing a delayed fixation of the assay, even 6 d.p.i. is adequate when the assay is performed in 6-well plates, since the plaques increase in size considerably at 6 d.p.i. When using the BHK cell line for FFU assays, fixation at 3 or 4 d.p.i. is recommended to ensure optimal foci definition and accurate quantification. Under these conditions, overlay concentrations of 0.2% and 0.4% are considered appropriate, as foci progressively enlarge between 3 and 4 d.p.i., facilitating their visualization. For assays performed in 24-well plates, it is advisable to use an endpoint of 3 d.p.i., when the foci are smaller and more manageable for accurate quantification.

In contrast, despite prior reports suggesting plaque formation in Vero cells at 8 d.p.i. (as noted in the Arbovirus Catalog [3]), we did not observe plaque formation at 3, 4, 6 d.p.i. under any of the three low-melting agarose overlay concentrations tested. This suggests that the Vero cells may not support plaque formation for either CPCV strain. FFUs were also absent at 3 d.p.i., but small FFUs began to appear at 4 d.p.i. and became more distinct at 6 d.p.i. across all overlay concentrations. These results indicate that Vero cells are not suitable for PFU assays for CPCV. However, FFU assays may still be feasible starting from 4 d.p.i. with optimal visualization achieved at 6 d.p.i. (Table 3). Even though it was possible to visualize FFU at a concentration of 0.8% low melting agarose, the concentration was also considered non-suitable for FFU assay due to the damage during the overlay removal even when performed carefully. Importantly, no notable cytopathic effect (CPE) or significant monolayer damage was observed at any of these time points.

### 3.3. Final Overlay Conditions

Once all conditions involving different overlay matrices, concentrations and cell lines were examined, a comparative evaluation of PFU and FFU assays was performed. The assessment focused on the clarity, definition, and ease of counting individual plaques and foci at their respective peak resolution times. Each condition was qualitatively ranked based on visual criteria, including edge sharpness, contrast, and background interference, as determined through empirical observation and practical experience acquired during assay optimization. We prioritized overlay conditions that consistently produced countable, well-circumscribed plaques or foci, with minimal monolayer disruption and high visual contrast. The final classification, presented in Table 4, reflects the most suitable combinations for reliable quantification of infectious CPCV particles using both titration methods.

Viral titers of both CPCV strains were determined in BHK cells at 3 and 4 d.p.i. using PFU assay (Supplementary Figure S1). No significant differences were observed between the various concentrations of methylcellulose and low melting point agarose used as overlay matrices in same strains. A comparable outcome was obtained for FFU quantification under the same conditions and time points (Supplementary Figure S2), further supporting the interchangeability of these overlays in this context. Data from 6 d.p.i. were excluded from the analysis due to technical limitations, including the absence of detectable foci, plaque quality and inconsistent assay performance. FFU titers in Vero cells at 4 and 6 d.p.i. revealed no statistically significant differences between methylcellulose and low melting point agarose overlays when comparing titers within the same CPCV strain (Supplementary Figure S3). Notably, FFU titers for the CPCV strain RP 962768 were consistently higher in Vero cells compared to BHK cells under equivalent conditions, suggesting a modest cell-line-dependent enhancement of infectivity. Data from 6 d.p.i. using 0.4% and 0.8% methylcellulose overlays were excluded due to technical limitations or suboptimal assay performance.

Under the optimized conditions, plaque and focus counts decreased in a log-linear fashion with each 10-fold dilution of inoculum, confirming quantitative behavior across several orders of magnitude. Re-titration of the same virus stocks on independent days produced concordant back-calculated titers within the expected experimental variation, indicating internal consistency of the assay. This log-linear behavior across serial dilutions is intrinsic to plaque- and focus-forming assays; this is reflected in the titers already presented in Table 2 and Table 3 and Supplementary Figures S1–S3 is therefore not shown separately to avoid redundancy.

## 4. Discussion

The establishment of robust and standardized methodologies for viral quantification is crucial for understanding the biology, replication kinetics, and pathogenesis of emerging arboviruses. This study addresses a significant methodological gap in CPCV research by optimizing PFU and assays in different cell lines under various overlay conditions. The findings not only provide a reproducible framework for CPCV titration but also contribute to broader virological studies.

BHK cells demonstrated consistent plaque and focus formation under defined overlay conditions, making them a reliable choice for CPCV quantification. This aligns with prior studies where BHK cells were shown to be suitable for titration of several arboviruses, including yellow fever virus (YFV), Rocio virus (ROCV), Japanese Encephalitis virus (JEV), and Dengue virus (DENV) In contrast, Vero did not support CPCV plaque formation under any overlay condition, even with extended incubation, although FFU detection confirmed infection, while ArboCat reports plaque formation in Vero cells at 8 d.p.i. [3]. Indeed, senior members of our group have observed that Vero E6 and Vero 76 cells support plaque formation more readily than Vero CCL-81 for other orthoflaviruses, consistent with published reports showing that replication efficiency and plaque morphology differ across Vero sublines [24]. Future studies should explore alternative Vero cell lines to determine whether specific sublineages are more conducive to CPCV plaque formation.

Despite their inability to support PFU assays, Vero cells proved permissive to CPCV infection, as focus formation was successfully observed at 6 d.p.i. This suggests that FFU assays remain a viable method for quantifying CPCV in this cell line, as has been demonstrated for other orthoflaviviruses such as dengue virus (DENV) and West Nile virus (WNV) [25,26,27]. Additionally, the observed differences in FFU visualization at 3, 4, and 6 d.p.i. between both cell lines highlight the importance of optimizing time points based on virus-specific replication in different cell lines. FFU assays remain feasible in Vero, with optimal visualization at later time points, whereas in BHK earlier fixation avoids monolayer damage.

For PFU assays, the choice of overlay significantly impacted plaque formation. While both 0.2% and 0.4% agarose overlays were effective at supporting plaque development at 3–6 d.p.i., 0.8% agarose was found to be suboptimal due to difficulties in overlay removal without disturbing the monolayer. Similar findings have been reported for other viruses, where higher concentrations of agarose or methylcellulose can hinder proper plaque resolution [28]. Because no CPCV-specific infectivity standards or published CPCV PFU/FFU protocols were available, we verified quantitative performance by serial-dilution log-linearity and repeated re-titration of virus stocks across days and conditions, approaches recommended when external standards are unavailable. Our study also demonstrated that lower concentrations of methylcellulose (0.2% and 0.4%) are more susceptible to plate disturbances during incubation, while 0.8% methylcellulose provides better stability, mitigating well-to-well inconsistencies. From a practical standpoint, the use of methylcellulose as an overlay matrix offers a notable economic advantage over low melting point agarose, as it is substantially less expensive while yielding comparable results in terms of viral plaque and focus resolution. Although both matrices supported consistent CPCV quantification across cell lines and time points, the cost-effectiveness of methylcellulose may be a relevant consideration for laboratories aiming to optimize large-scale screening workflows or routine viral titrations.

Both CPCV strains behaved comparably overall, though minor morphological differences were noted, likely reflecting intrinsic replication kinetics. These subtle phenotypic differences may hint at distinct viral fitness profiles, which could be associated with strain-specific replication rates, cytopathicity, or host cell interactions. While further investigation is needed, such preliminary distinctions could inform future studies on CPCV pathogenesis and in vitro/in vivo virulence.

Future studies should explore whether these differences translate to phenotypic divergence in vivo and in vitro. Moreover, adapting these protocols to mosquito-derived cell lines may aid in studies on vector competence and transmission dynamics. Although optimized using cell culture supernatants, these PFU and FFU conditions can also be adapted to quantify virus from infected serum or tissue homogenates after clarification steps to remove debris or cytotoxic components. In addition to vector competence applications, these standardized PFU and FFU assays could also be adapted for antiviral drug screening platforms. Their reproducibility, scalability, and compatibility with different overlay systems make them suitable for medium-to-high-throughput workflows aimed at evaluating candidate compounds that inhibit CPCV replication. Such translational uses extend the value of these protocols beyond basic virological research, supporting future therapeutic development and outbreak preparedness strategies. Although this study was not designed to benchmark CPCV titration against alternative approaches such as TCID_50_ or qRT-PCR, the reproducible PFU and FFU conditions presented here establish the necessary foundation for future cross-validation studies. A limitation of this study is that external cross-validation with alternative techniques such qRT-PCR was not systematically performed. This reflects the absence of CPCV-specific reference standards and the scope of this work as a methodological baseline. Nevertheless, we performed a preliminary TCID_50_ assay in C6/36 cells (Supplementary Figure S4), which yielded titers comparable in magnitude to those obtained with PFU and FFU assays. These results provide supportive evidence but are not intended as a comprehensive validation, which should be addressed in future studies. Another limitation of this study is that the number of replicates per dilution was insufficient to formally calculate regression parameters of assay log-linearity. Instead, reproducibility was verified by repeatedly re-titrating the same viral stocks across overlays, cell types, and incubation times, consistently yielding concordant titers (Table 2 and Table 3). While this provides internal validation of the quantitative reliability of the assay, larger datasets will be required to systematically assess log-linear regression parameters. Such analyses, including those generated by collaborating laboratories applying these standardized protocols, will provide external corroboration and further strengthen the robustness of CPCV titration methods.

## 5. Conclusions

In conclusion, this study represents a significant advancement in the virological characterization of CPCV by establishing and standardizing two assays—plaque-forming assay and focus-forming assay—which were previously unavailable in the literature for this virus. The identification of optimal overlay conditions, cell lines, and time points for these assays provides a robust framework for accurately quantifying viral infectivity and assessing cytopathic effects. This work not only fills a critical methodological gap but also lays the groundwork for future experimental studies aiming to elucidate CPCV’s replication dynamics, pathogenesis, and interactions with host cells. The careful evaluation of overlay concentrations and time points ensures reproducibility and reliability, making these assays valuable tools for high-resolution virological studies. The observed plaque morphology and focus size formation provide essential insights into the virus’s cytopathic profile, highlighting its potential to disrupt host cell monolayers through unique mechanisms.

These assays are particularly critical for future research aiming to evaluate antiviral compounds, study viral fitness, and conduct phenotypic assessments of CPCV mutants. By enabling precise viral titration and infection quantification, these methodologies are indispensable for advancing our understanding of CPCV biology and its potential role in emerging zoonotic threats. In conclusion, we present the first standardized plaque- and focus-forming assays for CPCV. By defining optimal overlay conditions, cell lines, and incubation times, this work provides a reproducible framework for quantifying CPCV infectivity. While preliminary, these protocols address a critical methodological gap and lay the foundation for future studies of CPCV biology, pathogenesis, and antiviral testing.

## Figures and Tables

**Table 1:** Overlay conditions used for protocol optimization.

Overlay Condition	Concentration
Methylcellulose	0.2%, 0.4% or 0.8%
Low melting Agarose	

**Table 2:** Range of Plaque and Focus Sizes in Vero and BHK Cells Using Different Methylcellulose Overlay Conditions.

	BHK						Vero					
**Strain BeAn 327600**												
	PFU			FFU			PFU			FFU		
	0.2%	0.4%	0.8%	0.2%	0.4%	0.8%	0.2%	0.4%	0.8%	0.2%	0.4%	0.8%
3 d.p.i.	1.10–2.58	0.87–2.53	0.56–2.50	1.43–2.93	1.66–2.95	1.45–3.22	n/o	n/o	n/o	n/o	n/o	n/o
4 d.p.i.	n/m	1.61–5.14	0.75–3.14	n/m	2.27–4.85	1.41–3.44	n/o	n/o	n/o	0.29–0.49	0.26–0.43	0.22–1.03
6 d.p.i.	n/m	n/m	n/m	n/m	n/m	n/m	n/o	n/o	n/o	0.36–5.23	n/m	n/m
**Strain RP 962768**												
	PFU			FFU			PFU			FFU		
	0.2%	0.4%	0.8%	0.2%	0.4%	0.8%	0.2%	0.4%	0.8%	0.2%	0.4%	0.8%
3 d.p.i.	0.77–2.57	1.17–2.21	0.54–2.18	1.51–4.31	1.35–3.32	1.84–3.03	n/o	n/o	n/o	n/o	n/o	n/o
4 d.p.i.	n/m	1.44–3.95	1.43–3.58	n/m	1.60–4.30	1.40–5.27	n/o	n/o	n/o	0.36–3.28	0.26–0.58	0.22–0.36
6 d.p.i.	n/m	n/m	n/m	n/m	n/m	n/m	n/o	n/o	n/o	3.35–4.70	n/m	n/m

All diameter measurements are in millimeters. n/o: plaques/foci not observed. n/m: not measured.

**Table 3:** Comparison of Plaque and Focus Sizes in Vero and BHK Cells Using Different Low Melting Agarose Overlay Conditions.

	BHK						Vero					
**Strain BeAn 327600**												
	PFU			FFU			PFU			FFU		
	0.2%	0.4%	0.8%	0.2%	0.4%	0.8%	0.2%	0.4%	0.8%	0.2%	0.4%	0.8%
3 d.p.i.	1.53–3.87	1.57–3.29	1.44–2.23	1.83–5.31	1.94–3.45	n/m	n/o	n/o	n/o	n/o	n/o	n/o
4 d.p.i.	1.64–5.69	1.74–4.72	1.82–4.00	2.91–5.57	2.51–5.57	n/m	n/o	n/o	n/o	0.52–3.80	0.46–3.71	n/m
6 d.p.i.	4.48–5.70	1.71–4.92	n/m	n/m	n/m	n/m	n/o	n/o	n/o	0.52–4.11	0.46–4.41	n/m
**Strain RP 962768**												
	PFU			FFU			PFU			FFU		
	0.2%	0.4%	0.8%	0.2%	0.4%	0.8%	0.2%	0.4%	0.8%	0.2%	0.4%	0.8%
3 d.p.i.	0.95–3.05	1.01–3.40	1.52–2.51	2.07–4.30	1.67–3.01	n/m	n/o	n/o	n/o	n/o	n/o	n/o
4 d.p.i.	0.83–5.28	1.71–4.24	1.05–3.58	3.56–5.12	2.10–4.78	n/m	n/o	n/o	n/o	1.95–4.97	0.75–5.48	n/m
6 d.p.i.	1.43–4.48	1.25–4.34	n/m	n/m	n/m	n/m	n/o	n/o	n/o	1.95–4.22	0.77–5.30	n/m

All diameter measurements are in millimeter. n/o: plaques/foci not observed. n/m: not measured.

**Table 4:** Comparative evaluation of CPCV plaque- and focus-forming assay performance under different overlay matrices, concentrations, cell lines, and incubation times.

Overlay Matrix	Concentration (%)	Cell Line	D.P.I.	PFU Assay Rating *	FFU Assay Rating *
Agarose	0.2	BHK	3	+++	++
			4	+++	++
			6	++	ns
	0.4		3	+++	++
			4	+++	++
			6	++	ns
	0.8		3	ns	ns
			4	ns	ns
			6	ns	ns
Methylcellulose	0.2		3	++	+
			4	ns	ns
			6	ns	ns
	0.4		3	+++	++
			4	++	+
			6	ns	ns
	0.8		3	+++	++
			4	+++	++
			6	ns	ns
Agarose	0.2	Vero	3	ns	ns
			4	ns	+
			6	ns	+++
	0.4		3	ns	ns
			4	ns	+
			6	ns	+++
	0.8		3	ns	ns
			4	ns	+
			6	ns	+++
Methylcellulose	0.2		3	ns	ns
			4	ns	+
			6	ns	+
	0.4		3	ns	ns
			4	ns	+
			6	ns	ns
	0.8		3	ns	ns
			4	ns	+
			6	ns	ns

* Legend: + = poor visibility; ++ = moderate; +++ = good; ns = not suitable (overlay condition produced indistinct, confluent, or uncountable plaques, rendering it unsuitable for reliable titration).

## Data Availability

All raw data described in this manuscript and Supplementary Data are available for access through Zenodo Data DOI 10.5281/zenodo.16543947.

## Supplementary Materials

The following supporting information can be downloaded at: https://www.mdpi.com/article/10.3390/v17101355/s1, Figure S1: Quantification of infectious titers of CPCV strains in BHK-CCL10 cells using distinct overlay matrices; Figure S2: Quantification of focus-forming units (FFU) of CPCV strains in BHK-CCL10 cells under distinct overlay conditions; Figure S3: Quantification of focus-forming units (FFU) by immunostaining of CPCV strains in Vero cells under distinct overlay matrices; Figure S4: Preliminary TCID_50_ assay of CPCV strains BeAn 327600 and RP 962768 performed in C6/36 cells at 5 dpi; Detailed Protocol: Preparing to Plaque-Forming Assay on BHK CCL-10 or Focus-Forming Assay on BHK CCL-10 or Vero CCL-81 Cells; Detailed Protocol: Immunohistochemistry of Fixed Monolayers.
