# Supplementary material for: Standardization of Quantitative Plaque-Based Viral Assays for Orthoflavivirus Cacipacoré
Source: Viruses. 2025 Oct 10;17(10):1355. doi: 10.3390/v17101355 (PMC12568257; doi:10.3390/v17101355)
Supplement: Supplementary file 1 [file viruses-17-01355-s001.zip › viruses-3815546-supplementary.pdf]

viruses

MDPI

Short Communication - Supplementary Materials

Standardization of Quantitative Plaque-Based Viral Assays for Orthoflavivirus Cacipacoré

Marielena Vogel Saivish [1,2,3], Natalia I. O. da Silva[3], Madeline R. Steck[3], Rafael E. Marques[2], Mauricio L. Nogueira[1,3], Shannan L. Rossi [3,5*] and Nikos Vasilakis [3,4,5,*]

1 Laboratórios de Pesquisas em Virologia, Departamento de Doenças Dermatológicas, Infecciosas e Parasitárias, Faculdade de Medicina de São José do Rio Preto, São José do Rio Preto, SP 15090-000, Brazil
2 Laboratório Nacional de Biociências, Centro Nacional de Pesquisa em Energia e Materiais (CNPEM), Campinas, SP 13083-100, Brazil
3 Department of Pathology, University of Texas Medical Branch, Galveston, TX 77555, USA
4 Center for Vector-Borne and Zoonotic Diseases, University of Texas Medical Branch, Galveston, TX 77555-0609, USA
5 Institute for Human Infection and Immunity, University of Texas Medical Branch, Galveston, TX 77555-0610, USA

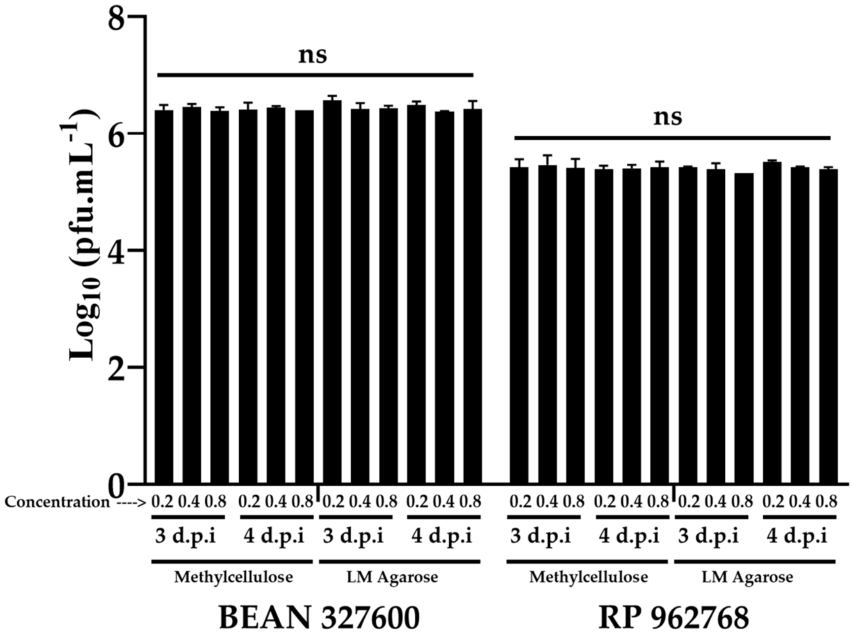

Supplementary Figure S1. Quantification of infectious titers of CPCV strains in BHK-CCL10 cells using distinct overlay matrices. Bar graphs display the viral titers ($Log_{10}(PFU.mL^{-1})$) of two distinct CPCV strains in BHK cells at 3 and 4 days post-infection (d.p.i.), assessed under two overlay conditions: low-melting-point agarose and methylcellulose. Infectious titers were determined by standard plaque assay. Each condition was performed in technical duplicates across two independent biological experiments. Data are presented as mean ± SD of log_{10}-transformed PFU values ($Y = log_{10}[Y]$). ns: no statistically significant differences were observed. Statistical analysis was conducted using GraphPad Prism v8.0.1 (table format: grouped), applying a two-way ANOVA (ordinary model, $\alpha = 0.05$) followed by Tukey's multiple comparisons test.

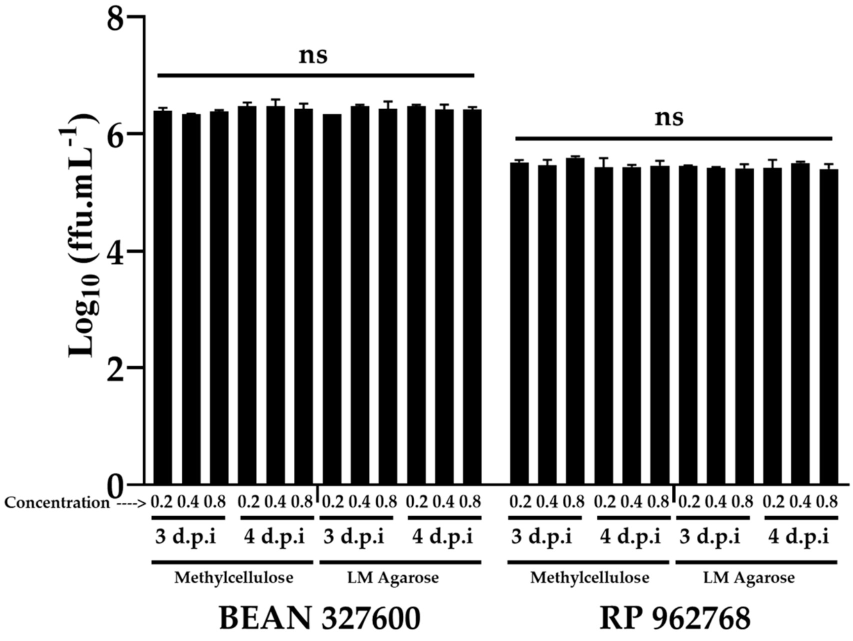

Supplementary Figure S2. Quantification of focus-forming units (FFU) of CPCV strains in BHK-CCL10 cells under distinct overlay conditions. Bar plots show the focus-forming unit (FFU) titers of two CPCV strains in BHK cells at 3 and 4 days post-infection (d.p.i.), using low-melting-point agarose or methylcellulose as overlay matrices. FFU titers were determined by focus assay. Each condition represents the mean ± SD of $\log_{10}$-transformed FFU values ($Y = \log_{10}[Y]$), derived from two independent biological experiments. No statistically significant differences (ns) were observed between overlay types within the same viral strain and time point. Statistical analysis was performed using GraphPad Prism v8.0.1 (grouped table format), applying a two-way ANOVA (ordinary model, $\alpha = 0.05$) followed by Tukey's multiple comparisons test.

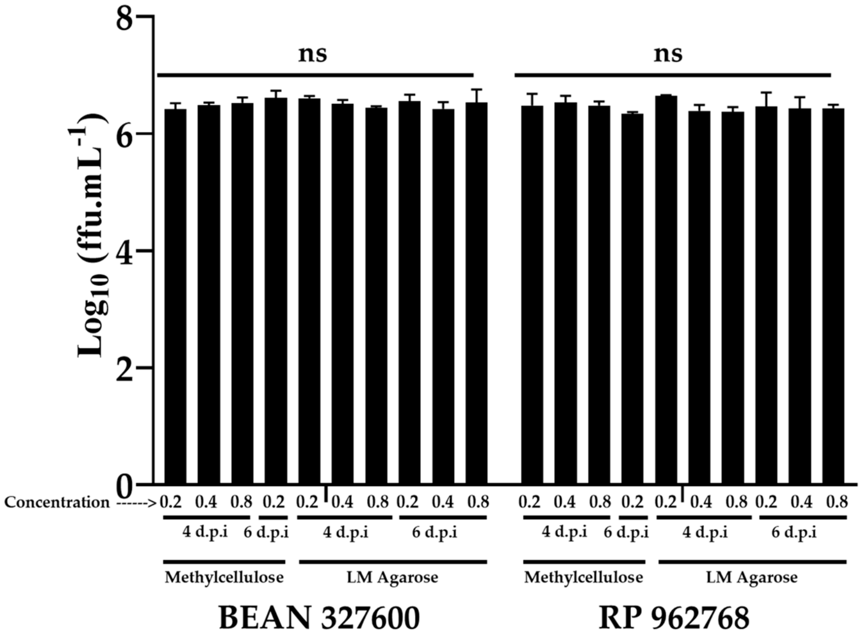

Supplementary Figure S3. Quantification of focus-forming units (FFU) by immunostaining of CPCV strains in Vero cells under distinct overlay matrices. Bar graphs depict the FFU titers of two CPCV strains in Vero cells CCL-81 at 4 and 6 days post-infection (d.p.i.), using either low melting point agarose or methylcellulose overlays. FFU titers were determined by immunostaining-based focus-forming assay. Each condition represents the mean ± SD of log_{10}-transformed FFU values (Y = log_{10}[Y]), derived from two independent biological experiments. No statistically significant differences (ns) were detected between overlay conditions within the same viral strain and time point. Statistical analysis was performed using GraphPad Prism v8.0.1 (grouped table format), applying a two-way ANOVA (ordinary model, $\alpha = 0.05$) followed by Tukey's multiple comparisons test. Only conditions with suitable foci morphology and quantifiable signal were plotted; other time points and overlays were excluded due to technical limitations, absence of detectable foci, or suboptimal assay performance.

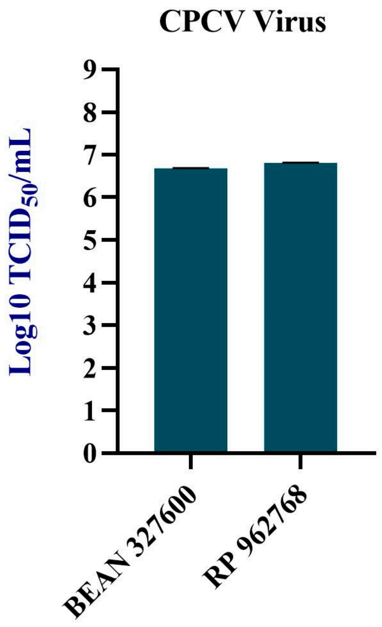

Supplementary Figure S4. Preliminary $TCID_{50}$ assay of CPCV strains BeAn 327600 and RP 962768 performed in C6/36 cells at 5 dpi. Bars represent mean log_{10} $TCID_{50}$/mL ± SD. Titers were in the same order of magnitude as those obtained with PFU/FFU assays, providing supportive but preliminary evidence of assay consistency. These results are not intended as systematic cross-validation, which remains a subject for future work.

Detailed Protocol: Preparing to Plaque-Forming Assay on BHK CCL-10 or Focus-Forming Assay on BHK CCL-10 or Vero CCL-81 Cells

1.0 Purpose

The purpose of this protocol is to provide a reproducible, step-by-step framework for quantifying infectious CPCV particles using either the plaque-forming assay (PFU) in BHK-21 CCL-10 cells or the focus-forming assay (FFU) in BHK-21 CCL-10 and Vero CCL-81 cells. These assays allow precise measurement of viral infectivity by visualizing virus-induced cytopathic effects (plaque forming units - PFU) or immunostained foci of infection (focus forming units - FFU) under defined overlay and incubation conditions. Establishing standardized procedures for overlay preparation, inoculation, fixation, and staining ensures that titration results are consistent across experiments and laboratories, facilitating studies of CPCV replication kinetics, host–virus interactions, and antiviral evaluation.

2.0 Methods

a. Seed 5×10^5 cells Vero CCL-81 or BHK CCL-10 cells in 6-well tissue culture plates in DMEM 10% FBS 1% penicillin/streptomycin (P/S) in a biosafety cabinet. Incubate cells in a 37°C incubator with 5% CO_2 for 1 day, until a nice monolayer of cells can be seen.
b. Thaw an aliquot of the virus cultured above and perform 10-fold serial dilutions in DMEM 2% FBS 1%P/S. We recommend doing at least 6 serial dilutions.

c. Aspirate and discard the cell media from wells. Infect the cells with 100 µL/well of the serially diluted virus in duplicates. Incubate for 1 h in 37°C incubator with 5% CO_2. Gently rock the plates from side to side, every 15 min, to ensure good contact between the virus and cells, and to prevent cells from drying out.
d. After 1 h absorption, discard the virus inoculum and replace with one of the overlay media described in the present manuscript (based on the results shown in the present manuscript, Section 3.3- PFU or FFU assay).
e. Incubate plates for 3, 4, or 6 days (according to the matrix chosen based on the results shown in the present manuscript, Section 3.3) in a 37°C incubator with 5% CO_2. There is no need to change the media at this step.
f. If doing PFU assay, incubate the plates with 10% formalin for 30 min to fix the cells and inactivate the viruses. If your aim is to perform the FFU assay, go directly to the instructions in the "**Detailed Protocol: Immunohistochemistry of Fixed Monolayers** " below.
g. Discard the supernatant by decanting or aspirating with a serological pipette. Rinse plate under running tap water.
h. Stain the cell monolayer with 1% crystal violet for approximately 15–30 min.
i. Wash the plate extensively under running tap water to visualize the plaques.
j. Allow the plates to air dry before counting the number of plaques to determine the virus titer in plaque forming unit per milliliter (pfu/mL)

Detailed Protocol: Immunohistochemistry of Fixed Monolayers

1.0 Purpose

The purpose of this protocol is to describe in detail the immunohistochemical detection of CPCV antigens in fixed cell monolayers using the pan-flavivirus monoclonal antibody 4G2. This assay forms the basis of the focus-forming unit (FFU) quantification method, enabling sensitive visualization of infected foci in the absence of overt cytopathic effect. By providing standardized conditions for fixation, antibody incubation, and chromogenic detection, this protocol ensures reproducible identification of CPCV-infected cells in both BHK-21 CCL-10 and Vero CCL-81 monolayers. Its adoption facilitates consistent viral titration, supports comparative infectivity studies, and allows integration of CPCV into broader orthoflavivirus research frameworks.

2.0 Materials and Equipment

- Serological pipettes
- PBS (non-sterile) from Gibco
- Primary antibody against the antigen. We use the Anti-Flavivirus Group Antigen Antibody, clone D1-4G2-4-15. MAB10216-I from Sigma; 100µg (1:500)
- Peroxidase-labeled secondary antibody against the species of the primary. We use the Anti-Mouse IgG (H+L) Antibody, Human Serum Adsorbed and Peroxidase-Labeled; Sera Care 5220-0286.
- A plate rocker
- A 1ml pipette with non-filtered tips
- DI water
- Blocking buffer (3% Fetal Bovine Serum [FBS]) in PBS
- Enzo peroxidase developing kit (AEC peroxidase substrate kit ENZ-43825) or KPL TrueBlue™ Peroxidase Substrate (5510-0030)

3.0 Requirements

3.1 Cells whose intracellular contents need to be assayed need to be lysed open either during or after the fixation process. A 50/50 mix of methanol and acetone for 30 minutes will pop open the cells. A 4% paraformaldehyde solution will not, and in this case will need to be treated with a detergent prior to the assay.

4.0 Procedure

NOTE: This procedure works best with Methanol:acetone-fixed plates, but can be adapted for immunofluorescent assays by altering the blocking buffers and secondary antibody.

4.1 Dry the plates after fixation. This can be done overnight on the bench or on the BSC grate when not in use.

4.2 Add blocking buffer to each well with an appropriate volume for the well (volumes described in Table 1 below). Place the plate to shake for at least 30 minutes.

4.3 Prepare enough primary antibody in blocking buffer to use on all wells. Since it was commercially purchased, it should come with a recommendation.

4.4 Dump the blocking buffer out into the sink, gently invert the plate and tap the excess media onto a paper towel.

4.5 Add the diluted primary antibody to each well as suggested below. Place plates on the rocker and incubate at room temperature for at least 2 hours. This can be done overnight if started late in the day.

4.6 Wash the wells 3 times with PBS at least 5 minutes/wash. Place on rocker in between washes. If the primary was done overnight, extend wash times to 15 minutes.

4.7 During the washes, dilute the peroxidase-labeled secondary as done for the primary in step 5.3. . Since it was commercially purchased, it should come with a recommendation.

4.8 After the last wash, dump out PBS and add secondary antibody diluted in blocking buffer made in step 5.7. Place back onto the rocker for at least one hour. This can go longer, but no more than 3 hours to avoid high background staining.

4.9 Wash wells 3 times with PBS as done in step 5.6.

4.10 As the last wash is on, create the developing buffer as per Enzo's instructions or use the KPL ready solution. This should be done in a 50 ml conical wrapped in tin foil to avoid exposure to the light. Calculate the volume needed to cover all wells.

4.11 After adding the developing agent, place plates in tin foil in the dark on the rocker. Check for the development of a pinkish color after about 5 minutes. If a faint pink is seen (in case of Enzo's kit; or faint blue in case of KPL kit), check again in 5 minutes. Do not exceed 30 minutes. If the background starts to appear pink, stop reaction.

4.12 Take the plates from the tin foil and wash the monolayers once with DI water. This stops the reaction and the plates can be read or imaged at this time.

Table S1. Suggested Volumes for blocking, antibody and developing. Volumes are suggestions. Be aware of the meniscus that will preferentially stain the perimeter of the wells if the volume is too low. Too much and you'll waste antibody.

Well size	Blocking volume	Antibody volume	Developing volume
6-well plate	1 ml	At least 300 μL	500 μL
12-well plate	500 μL	At least 250 μL	At least 300 μL
24-well plate	500 μL	At least 125 μL	At least 250 μL
Spot slide	20 μL/spot	20 μL/spot	20 μL/spot
